# Supply and Clinical Application of Actinium-225 and Bismuth-213

**DOI:** 10.1053/j.semnuclmed.2020.02.003

**Published:** 2020-03

**Authors:** Alfred Morgenstern, Christos Apostolidis, Frank Bruchertseifer

**Affiliations:** European Commission, Joint Research Centre, Directorate for Nuclear Safety and Security, Karlsruhe, Germany

## Abstract

The recent development of ^225^Ac-PSMA617 for therapy of prostate cancer has strikingly demonstrated the clinical potential of targeted alpha therapy. Further promising applications of the alpha emitters ^225^Actinium and its daughter nuclide ^213^Bismuth include the therapy of brain tumors, bladder cancer, neuroendocrine tumors, and leukemia. This paper will provide a brief overview on the current status of the clinical development of compounds labelled with ^225^Ac or ^213^Bi and describe the various production routes that are in place or are under development to meet the increasing demand for these radionuclides.

## Introduction

The concept of applying ^225^Actinium and its daughter nuclide ^213^Bismuth for targeted alpha therapy (TAT) of cancer was described by Geerlings et al in 1993.^1^ Although the nuclides were hardly accessible at that time, the authors recognized their potential for medical application, given their favorable decay characteristics and chemical properties combined with the inherent advantages of alpha radiation of high energy and short-range in human tissue. For two decades thereafter, extensive research was performed, including the development of methods for production of ^225^Ac and ^213^Bi, numerous preclinical studies and several clinical investigations. Early pioneering clinical work focused on treatment of leukemia,^2^^,^^3^ non-Hodgkins lymphoma (NHL),^4^ malignant melanoma,^5^, ^6^, ^7^ brain tumors,^8^, ^9^, ^10^, ^11^ neuroendocrine tumors,^12^^,^^13^ and bladder cancer.^14^ In 2013, 20 years after the initial work of Geerlings et al, the compound ^225^Ac-PSMA617 was first synthesized and investigated at Joint Research Centre (JRC) Karlsruhe. The remarkable efficacy of this novel compound manifested already in early patient studies conducted in collaboration of JRC and University Hospital Heidelberg and was first reported by Kratochwil et al in 2016.^15^ While the clinical application of ^225^Ac-PSMA617 was further developed in collaboration of JRC with hospitals in Heidelberg,^16^^,^^17^ Pretoria,^18^, ^19^, ^20^ and Munich,^21^ the remarkable benefit observed clinically in an increasing number of patients stimulated worldwide interest in applying ^225^Ac in TAT. Consequently, an increasing number of novel ^225^Ac-labeled compounds are currently under development, and production facilities to meet the increasing demand for the nuclide have been set up or are under construction. This paper will summarize important clinical investigations with ^225^Ac- and ^213^Bi-labelled compounds just briefly, as they will be described in much detail in other contributions in this issue. Furthermore, the current status of production of ^225^Ac will be reviewed.

## Clinical Experience With ^225^Ac and ^213^Bi

The current state of clinical investigation of ^225^Ac- and ^213^Bi-labelled compounds is summarized in Table 1. The early studies on TAT of leukemia, NHL, malignant melanoma, and the more recent pilot trial on bladder cancer investigated ^213^Bi- or ^225^Ac-labeled antibodies. While the combination of short-lived ^213^Bi (T_1/2_) = 46 minutes and full antibodies holds promise for the targeting of rapidly accessible, blood-associated cancers such as leukemia^2^^,^^3^ or NHL,^4^ the ^213^Bi-labelled anti-MCSP-antibody investigated for the treatment of malignant melanoma^5^, ^6^, ^7^ probably failed to deliver sufficient tumor dose due to the slower pharmacokinetics of the construct. In contrast, the intravesical application of ^213^Bi-anti-EGFR-mAb for therapy of carcinoma in situ^14^ is an excellent approach that facilitates rapid and very selective targeting and minimizes toxicity to other organs.

**Table 1:** Overview of Current Clinical Experience With ^225^Ac- and ^213^Bi-Labeled Compounds

Cancer Type	Radioconjugate	Patients	Reference
Leukemia	^213^Bi-anti-CD33-mAb	49	^2^^,^^3^
	^225^Ac-anti-CD33-mAb	76	^22^
Lymphoma	^213^Bi-anti-CD20-mAb	12	^4^
Melanoma	^213^Bi-anti-MCSP-mAb	54	5, 6, 7
Bladder cancer	^213^Bi-anti-EGFR-mAb	12	^14^
Glioma	^213^Bi-Substance P	68	8, 9, 10, 11
	^225^Ac-Substance P	20	^23^
Neuroendocrine tumors	^213^Bi-DOTATOC	25	^12^
	^225^Ac-DOTATOC	39	^13^
Prostate cancer	^225^Ac-PSMA617	>400	15, 16, 17, 18, 19, 20, 21

The combination of short-lived ^213^Bi and low molecular weight, rapidly diffusing peptides, such as DOTATOC and Substance P analogues, showed promising results for the treatment of neuroendocrine tumors^12^^,^^13^ and gliomas,^8^, ^9^, ^10^, ^11^ in particular after locoregional administration for improved tumor uptake. In collaboration of JRC Karlsruhe and University Hospital Heidelberg 25 patients with multiresistant neuroendocrine tumors refractory to therapy with beta emitter labeled ^90^Y-/^177^Lu-DOTATOC were treated with ^213^Bi-DOTATOC. Although patients presented in clinically challenging situations and had developed resistance against therapy with beta emitters, TAT with ^213^Bi-DOTATOC resulted in a high number of long-lasting antitumor responses, including one complete remission. Hematotoxicity was dose-limiting, and potential long-term renal toxicity has to be considered and closely monitored.

Locoregional application of ^213^Bi-[Thi^8^,Met(O_2_)^11^]-substance P for treatment of gliomas has been found safe after injection of doses of 2 GBq in 2-month interval up to cumulative doses of 11.2 GBq with only mild and transient adverse reactions. The median overall survival after recurrence of 10.9 months observed in a cohort of 20 patients with recurrent glioblastoma (GBM) compares favorably to available alternative treatments.^11^ Also in a small cohort of nine patients suffering from secondary GBM, treatment with ^213^Bi-[Thi^8^,Met(O_2_)^11^]-substance P was well tolerated and resulted in median overall survival of 18.6 months after conversion of the primary low grade glioma to grade IV GBM.^10^ Two out of nine patients with secondary GBM were still alive 39 and 51 months after initiation of locoregional alpha therapy.

While the combination of low molecular weight, rapidly diffusing peptides with local modes of administration holds promise for therapeutic application of short-lived ^213^Bi, the high cost and currently still limited supply of high activity ^225^Ac/^213^Bi generators required for preparation of patient doses in the multiple GBq range still poses considerable limitations for clinical application of these treatment approaches. These limitations can be overcome to some extent through application of ^225^Ac with its longer half-live of 9.9 days and its higher cytotoxicity associated with the multiple alpha particles generated in its decay chain.

The first in human clinical investigation of a ^225^Ac-labeled small molecule was started in 2011 in collaboration of JRC Karlsruhe and University Hospital Heidelberg for therapy of patients with neuroendocrine tumors using ^225^Ac-DOTATOC.^13^ In a group of 39 patients, the treatment was found to be safe with activities of 18.5 MBq given in 2-month interval up to a cumulative dose of 75 MBq. The MTD of a single dose was determined as 40 MBq. Although several of patient responses were observed, further investigations are needed to improve patient selection and fractionation regimes.

The development of ^225^Ac-PSMA617 for therapy of prostate cancer can be considered a milestone in the evolution of TAT. The pharmacokinetics of PSMA617, in particular its rapid tumor uptake within a few hours, its extended tumor retention as well as its rapid renal clearance of unbound compound provide an excellent match to the decay characteristics of long-lived ^225^Ac. In particular the high degree of internalization of PSMA617 allows intracellular capture of the decay daughters of ^225^Ac and utilizing their cytotoxicity for tumor cell kill, while minimizing toxicity of errant daughters. Following the development and in vitro characterization of ^225^Ac-PSMA617 at JRC Karlsruhe in 2013/2014, clinical testing was started initially in collaboration of JRC Karlsruhe with University Hospital Heidelberg, and subsequently extended to Steve Biko Academic Hospital Pretoria and Technical University Munich.

For initial clinical application of ^225^Ac-PSMA617, a standardized protocol was developed based on a dosimetry estimate and retrospective evaluation of the efficacy and tolerability of salvage therapies performed in 14 advanced stage metastatic castration-resistant prostate cancer patients, consisting of a treatment activity of 100 kBq/kg of ^225^Ac-PSMA617 per cycle repeated twice every 8 weeks.^16^ In a cohort of 40 advanced stage patients the protocol demonstrated remarkable antitumor efficacy, while xerostomia (dry mouth syndrome) was observed as main adverse effect leading to discontinuation of treatment in 10% of patients.^17^ In subsequent clinical investigations, ^225^Ac-PSMA617 was administered in fixed activities of 8 MBq for the first cycle (corresponding to 100 kBq/kg body weight in a typical patient of 80 kg) for reasons of standardization and simplification of procedures. In addition, to minimize damage to salivary glands, administered activities in subsequent cycles were de-escalated to 7-4 MBq as function of patient response.^18^ An analysis of 73 patients treated with ^225^Ac-PSMA617 at Steve Biko Academic Hospital in Pretoria demonstrated excellent therapeutic efficacy and increased tolerability of this de-escalation approach, showing a Prostate specific antigen (PSA) decline of ≥50% in 70% of patients, while 83% of patients had any PSA decline and 0% of patients discontinued treatment.^19^ Another promising approach for maintaining high therapeutic efficacy while minimizing toxicity to salivary glands is the combination of alpha- and beta emitter labelled PSMA617. An interim analysis of a first group of 17 patients with advanced metastatic castration-resistant prostate cancer treated with a combination of 4 MBq ^225^Ac-PSMA617 and 4 GBq ^177^Lu-PSMA617 demonstrated improved tolerability with a response rate of 76% (PSA decline >50%).^24^ Notably, therapy with ^225^Ac-PSMA617 was also found to be effective for brain metastases in advanced prostate cancer patients.^20^

## Production of ^225^Ac and ^213^Bi

### Radiochemical Extraction of ^225^Ac From ^229^Th

The radiochemical extraction of ^225^Ac from ^229^Th (T_1/2_ = 7917 years) sources has been the workhorse for production of ^225^Ac and ^213^Bi since the early 1990s. All clinical investigations and virtually all preclinical research with ^225^Ac and ^213^Bi-labelled compounds to date have been conducted with radionuclides obtained from ^229^Th decay. Worldwide three sources of ^229^Th are available that allow the production of clinically relevant activities of ^225^Ac/^213^Bi, located at the Directorate for Nuclear Safety and Security of the JRC of the European Commission in Karlsruhe, Germany (formerly known as *Institute for Transuranium Elements*),^25^ Oak Ridge National Laboratory (ORNL), USA^26^ and at the Institute of Physics and Power Engineering (IPPE) in Obninsk, Russia.^27^ The ^229^Th sources have been obtained by separation from aged, fissile ^233^U originally produced for weapons applications by neutron irradiation of natural ^232^Th. More recently the preparation of a small scale ^229^Th source has been also reported from Canadian Nuclear Laboratories.^28^ Notably, it has been reported that from 2019 onward a very significant increase in availability of ^229^Th will be generated through extraction of ^229^Th from legacy wastes stored within the US Department of Energy. The total amount of accessible ^229^Th has been estimated to up to 45 g, corresponding to a potential increase in ^225^Ac supply of up to 40 fold compared to current levels.^29^

The production of ^225^Ac from ^229^Th at JRC Karlsruhe is based on a combination of anion exchange and extraction chromatography,^25^^,^^30^ while the process in place at ORNL is utilizing anion and cation exchange.^26^ JRC has been the first laboratory to prepare ^225^Ac/^213^Bi for clinical use in the mid 1990s and has since then produced approximately 13 GBq ^225^Ac annually for preclinical research and clinical testing performed at JRC Karlsruhe or in collaboration with a wide network of clinical partners. The maximum annual production of ^225^Ac at ORNL is approximately 33 GBq, while IPPE has reported a production of 22 GBq per year.^27^ While ^225^Ac produced at JRC Karlsruhe and ORNL has been extensively applied for patient treatment and found safe for administration to humans, direct clinical application of ^225^Ac produced at IPPE to our knowledge has not been reported to date.

^225^Ac can be used directly as therapeutic nuclide or can be loaded on ^225^Ac/^213^Bi generators to provide short-lived ^213^Bi on site. In general ^225^Ac/^213^Bi generators based on AG MP-50 cation exchange resin are most established and have been used for all patient studies with ^213^Bi to date.^31^ The high activity generator system developed at JRC Karlsruhe allows reliable operation of the generator when loaded with activities up to 4 GBq ^225^Ac with yields of ^213^Bi elution exceeding 80% and a low breakthrough of ^225^Ac parent nuclide of less than 0.2 ppm (activity).^32^ A key feature during preparation of this generator is the process of loading the parent nuclide, resulting in the distribution of ^225^Ac activity over approximately two thirds of the generator resin in order to minimize radiolytic degradation of the organic resin and to assure reliable operation over several weeks. The generator has been successfully used clinically for preparation of therapeutic doses of ^213^Bi-labelled peptides with activities up to 2.3 GBq at time of injection.

### Accelerator-Based Routes

Currently the global supply of ^225^Ac from ^229^Th is still insufficient for widespread routine application of ^225^Ac and ^213^Bi-labelled compounds in hospitals worldwide. Consequently, a variety of alternative methods for large scale production of ^225^Ac have been investigated, including the spallation of ^232^Th or ^nat^U targets with highly energetic protons and the irradiation of ^226^Ra targets using protons, deuterons or gamma-rays.^33^

Among these routes, the production of ^225^Ac by spallation of ^232^Th is currently the most advanced. The feasibility of the process has been demonstrated at the Institute for Nuclear Research, Russian Academy of Sciences, in Troitsk, Russia^34^^,^^35^ and at Los Alamos National Laboratory in the US.^36^^,^^37^ In the last years the routine production of ^225^Ac *via* spallation of ^232^Th has been successfully established within the US DoE Tri-Lab (ORNL, BNL, LANL) effort. Irradiations can be performed at Brookhaven National Laboratory (200 MeV at 165 μA) and Los Alamos National Laboratory (100 MeV at 275 μA), while targets are processed and the final product is distributed from ORNL.^38^ The main limitation of the process is the coproduction of long-lived ^227^Ac (T_1/2_ = 21.8 years) at levels of 0.1%-0.2% activity (at EOB). Since ^225^Ac and ^227^Ac isotopes cannot be chemically separated, the implications of the ^227^Ac impurity for the clinical application of the ^225^Ac product have to be considered. Initial studies indicate that the impact of the ^227^Ac impurity on patient dosimetry will be negligible.^39^ However, issues related to licensing, safe handling, and disposal of long lived ^227^Ac in hospital settings still have to be resolved.

The production of ^225^Ac by proton irradiation of ^226^Ra targets in a cyclotron through the reaction ^226^Ra(p,2n)^225^Ac offers a number of advantages over the ^232^Th spallation reaction. The process can be performed with high yields in a cost-effective manner in medium-sized cyclotrons at proton energies below 20 MeV.^40^ A 24 hour irradiation of 50 mg ^226^Ra at the maximum of the excitation function at 15-16 MeV with a current of 100 µA protons is expected to yield approximately 5 GBq ^225^Ac, equivalent to 500 patient doses of 10 MBq ^225^Ac. Chemical purification of the irradiated targets yields ^225^Ac of high isotopic purity as no other long-lived actinium isotopes such as ^227^Ac (T_1/2_ = 21.8 years) are co-produced. Co-production of impurities of short-lived ^226^Ac (T_1/2_ = 29 hours) and ^224^Ac (T_1/2_ = 2.9 hours) formed according to the reactions ^226^Ra(p,n)^226^Ac and ^226^Ra(p,3n)^224^Ac can be minimized through selection of appropriate proton energies. In addition, their activity will further decay to low levels during the time required for target cooling and reprocessing. The main challenges of the process are related to the preparation and handling of targets containing milligram amounts of radioactive ^226^Ra (T_1/2_ = 1600 years) and management of its gaseous decay product ^222^Rn (T_1/2_ = 3.8 days). Currently research on implementation of this production route is ongoing in facilities in North and South America, Europe, and Asia.

The irradiation of ^226^Ra with deuterons according to the reaction ^226^Ra(d,3n)^225^Ac has been suggested as an improved method for production of ^225^Ac.^33^ Cross-sections for the reaction have not been measured experimentally yet, however, model calculations are predicting a maximum cross-section of 864 mb at 18.5 MeV and slightly increased production yields compared to the ^226^Ra(p,2n)^225^Ac reaction. However, deuteron irradiation would lead to an enhanced co-production of ^226^Ac (T_1/2_ = 29 hours) via the reaction ^226^Ra(d,2n)^226^Ac, making an extended cooling time necessary to allow for ^226^Ac decay. In addition, only few accelerators are available that can provide deuteron beams of sufficient energy.

Another route for production of ^225^Ac via irradiation of ^226^Ra is based on the photonuclear reaction ^226^Ra(γ,n)^225^Ra and subsequent beta decay of ^225^Ra to ^225^Ac. The reaction has a photon energy threshold of 6.4 MeV, experimentally measured cross-section data are not available to date. However, as modeling data predict moderate reaction yields,^41^ modern accelerators providing high-intensity electron beams are required for commercially viable production. Proof of principle of the process has been demonstrated on laboratory scale at JRC Karlsruhe.^42^ Betatron irradiation of a zircaloy capsule containing 1 mg ^226^Ra embedded in 800 mg BaCl_2_ matrix for 3.5 hours at 52 MeV led to production of 0.24 µCi of ^225^Ac. Feasibility of the process has also been successfully demonstrated at Joint Institute for Nuclear Research, Dubna, Russia^43^ and at the Illawarra Cancer Centre, in Wollongong, Australia.^44^ Maslov et al reported a radiation yield of 550 Bq/(µAh mg ^226^Ra) at 24 MeV maximal photon energy. The detailed measurement of cross-section data for this reaction is highly desirable for more accurate prediction of production yields. Nevertheless, implementation of the process for large scale production of ^225^Ac is already underway at several facilities.^45^

## Conclusions

Reports on the remarkable therapeutic efficacy of ^225^Ac-PSMA617 for therapy of prostate cancer have stimulated significant global interest in applying ^225^Ac as therapeutic nuclide in TAT of cancer. It is expected that in order to meet the rising future demand for supply of the alpha emitter a variety of production routes will be utilized, including the extraction of additional ^229^Th stocks from US legacy wastes and the implementation of various accelerator based routes. In this respect common criteria for quality of the ^225^Ac product need to be established in order to facilitate safe clinical use of the radionuclide independent of its production pathway.
